# Solan on the Pommade of Oxide of Zinc in Eczema, Impetigo, and Ecthyma

**Published:** 1842-10-15

**Authors:** Martin Solan


*Pommade of Oxide of Zinc in Eczema, Impetigo, and Ecthyma.* By Martin Solan.—In the above disease Martin Solan has experienced great success from the employment of the following ointment: Axungiæ, 30 grammes; Oxidii Zinci Alb., 1 vel 3. M. This ointment to be rubbed on the affected part morning and evening, with a sufficient quantity of pomatum. The three grammes of the oxide give the compound considerable consistence. It should not be employed until desquamation has been established and all irritation removed, no matter whether eczema extends over a large portion of the body, or be confined to the backs of the ears, to the armpits, to the bend of the elbow, wrist, or knee; in either case it is *seldom this medicine fails* in removing the itching, in diminishing the redness and local secretion, and affecting a speedy cure, particularly if combined with baths.

In some cases of chapped nipple, or slight excoriations of the vulva, this pommade has been found extremely useful. Impetigo and ecthyma have yielded to it with equal rapidity. We ought here, as in every other instance, to employ, along with the local treatment, means calculated to improve the general health.—*Lond. Med. Gaz., from Journal des Connaisances Médico-Chirurgicales*, June, 1842.

